# Activation Markers on B and T Cells and Immune Checkpoints in Autoimmune Rheumatic Diseases

**DOI:** 10.3390/ijms23158656

**Published:** 2022-08-04

**Authors:** Elena V. Gerasimova, Dmitry V. Tabakov, Daria A. Gerasimova, Tatiana V. Popkova

**Affiliations:** 1 Department of Systemic Rheumatic Diseases, V.A. Nasonova Research Institute of Rheumatology, Kashirskoe Shosse, 115522 Moscow, Russia; 2 Department of Organization and Economy of Pharmacy, Institute of Pharmacy, I.M. Sechenov First Moscow State Medical University (Sechenov University), 8/2, Trubetskaya St., 119526 Moscow, Russia

**Keywords:** immune checkpoints, B-cell, T-cell, autoimmune rheumatic diseases, targeted immunotherapy

## Abstract

In addition to identifying the major B- and T-cell subpopulations involved in autoimmune rheumatic diseases (ARDs), in recent years special attention has been paid to studying the expression of their activation markers and immune checkpoints (ICPs). The activation markers on B and T cells are a consequence of the immune response, and these molecules are considered as sensitive specific markers of ARD activity and as promising targets for immunotherapy. ICPs regulate the activation of the immune response by preventing the initiation of autoimmune processes, and they modulate it by reducing immune cell-induced organ and tissue damage. The article considers the possible correlation of ICPs with the activity of ARDs, the efficacy of specific ARD treatments, and the prospects for the use of activation molecules and activation/blocking ICPs for the treatment of ARDs.

## 1. Introduction

Autoimmune rheumatic diseases (ARDs) are a heterogeneous group of disorders characterized by a self-sustaining autoreactive adaptive immune response, leading to immune-mediated damage to target organs. The etiology of these diseases is still unknown, and at the moment, it is thought that ARDs develop from a set of poorly defined interactions between environmental triggers and polymorphic genomic elements, leading to the loss of immunological tolerance and the development of autoimmune inflammation [1].

In recent years, remarkable advances have been made in cell-type phenotyping and the understanding of intercellular interaction mechanisms, intracellular signaling pathways, and genetic control of the immune system. The discovery of the fact that the T- and B-cell response to the antigen (autoantigen) is regulated by a combination of various factors has led to the study of non-antigenic determinants of autoimmunity. The studies discuss the activation markers in the B- and T-cell relationship with the pathogenesis and the degree of ARD activity and show the promise of immunotherapeutic blocking and activating drugs with these molecules.

## 2. B- and T-Cell Activation in Autoimmune Rheumatic Diseases

It is known that the pathogenesis of ARDs is based on the loss of B-cell tolerance to its own antigens as a result of a defect in the negative selection of autoreactive B-cell clones. It leads to the polyclonal activation of B lymphocytes and the hyperproduction of a wide range of autoantibodies (autoAbs) [2,3]. In addition, B cells induce non-antibody-dependent reactions: the pathological activation of the T-cell component of the immune response, the differentiation of follicular dendritic cells (DCs), and the synthesis of cytokines. It is believed that DCs perform the function of antigen-presenting cells (APCs) at an early stage of the immune response and “memory” B cells at a later chronic stage of autoimmune inflammation. Memory B cells express the CD27 molecule, which promotes the differentiation of activated memory B cells into plasma cells by interacting with the T-cell ligand (CD70). On the surface of B lymphocytes, autoantigens in combination with the molecules of the major histocompatibility complex (MHC) of class II are recognized by the T-cell receptor (TCR). This interaction, along with the expression of the co-stimulating molecules, provides optimal conditions for the activation, proliferation, and survival of autoreactive CD4+ T cells.

Despite fact that B cells play a key role in the pathogenesis of ARDs, their response to self-antigen needs help from the T-cell compartment. T cells play a central role in the mechanisms of tolerance, which makes their participation in the development of immune disease necessary and decisive. Maintaining peripheral tolerance depends on the fact that T cells encounter their own antigen in a non-immunogenic context. The main factor determining the immune response to the antigen is the involvement of co-stimulating receptors along with the TCR-mediated detection of the antigen. The difference between the tolerant immunogenic interactions with an antigen is based on the increased expression of the co-stimulating molecules and the activation of the co-stimulating signals. Thus, in addition to the direct effect on the B lymphocytes, the actual goal is to change the level of T-cell stimulation by purposefully influencing the co-stimulating pathways [4,5]. 

The detection of T-cell oligoclonality and B-cell hypermutation in synovial tissue at the preclinical stage of the disease may indicate the aberrant activation of the adaptive immune system contributing to the occurrence of RA [6].

According to modern concepts, activated CD4+ T lymphocytes play a key role in the development of synovial inflammation and joint destruction in RA. Their interaction with autoantigens leads to the activation of B lymphocytes and macrophages, as well as to increased cytokine production [7,8]. The detection of circulating CD4+ T cells with phosphorylated proteins p38—mitogen-activated protein kinase (MAP), transcription factors c-Jun (a protein that in humans is encoded by the JUN gene), and NF-kB (nuclear factor kappa B)—allows the differentiation of early RA patients from healthy individuals [9].

The role of activated blood CD4+ T lymphocytes in patients with systemic lupus erythematosus (SLE) has been less studied. It is known that the inflammatory epigenetic transformations of naive CD4+ T cells in SLE patients precede transcription changes and can balance naive CD4+ T cells for the immune responses of T helper (Th) 2/Th17/T follicular helper cells (Tfh) [10].

In recent years, data have been obtained on the ability of T regulatory cells (Tregs) to suppress various immune-inflammatory responses under the influence of a wide range of physiological and pathological stimuli [11]. Tregs express a wide range of membrane molecules that determine their functional activity and make it possible to identify these cells in the bloodstream [12], but there is still controversy about a universal surface marker that makes it possible to isolate this cell subpopulation from the pool of T lymphocytes.

Tregs produce interleukin (IL)-10 and transforming growth factor-beta (TGF-β) [13]. The loss of the ability of Tregs to inhibit the synthesis of IL-6 and interferon (IFN)-γ by effector cells, while maintaining the ability to limit the proliferation of these cells, has been demonstrated in experimental models and ARD patients [11]. The suppression of Treg pathways, such as signaling by transforming growth factor beta (TGF-β), disrupts the cellular differentiation and other functions of most immune cells [10,14]. These changes in the cytokine landscape can disrupt the normal immune landscape and suppress the regulatory T-cell pathways [15].

Currently, in addition to determining the main populations of lymphocytes, more and more attention is being paid to identifying the activation markers of the B- and T-cell links of the immune system. It has been shown that the expression of the surface markers of B and T lymphocytes correlates with the degree of ARD activity and has a prognostic value in evaluating the effectiveness of therapy [16,17].

## 3. Prognostic Role of Surface Molecules on B and T Cells in Autoimmune Rheumatic Diseases

To date, the role of lymphocyte activation receptors in the pathogenesis and prognosis of ARDs has been established. The greatest attention is paid to the expression of CD95, HLA-DR, and CD25 on various subpopulations of lymphocytes, both normal and pathological, and their connection with the development of ARDs.

### 3.1. CD95 

CD95 (also called Fas) is a member of the tumor necrosis factor (TNF) receptor superfamily. The interaction of Fas with its CD95L ligand is involved in the processes of ensuring homeostasis of the immune system and immune surveillance. Fas and FasL play complex roles in the immune system. Present in a large variety of cells, the regulation of their expression and the consequences of their engagement vary greatly depending on the cell context in which they appear. Although Fas is constitutively expressed on the surface of most activated T cells, only effector memory T cells are highly sensitive to Fas-induced cell death. The expression of Fas and FasL is differently induced on CD8 T cells depending on the conditions. Thus, a high expression of Fas and FasL, induced during presentation of the autoantigen, can regulate CD8 expression and cell survival and, therefore, contribute to the regulation of the T-cell responses to autopeptides [18]. It is known that mutations in this receptor are associated with the loss of the apoptotic signal and have been found in patients with autoimmune disorders, particularly in type 1a Autoimmune Lymphoproliferative Syndrome, which has similar clinical manifestations to SLE [19]. Links have been found between CD95 or its apoptotic mechanism, including the adapter protein, the Fas-associated death domain (FADD) protein, and the protease initiator caspase-8, in the activation/regulation of damage-associated molecular pattern (DAMP) complexes [20].

**Figure 1:**
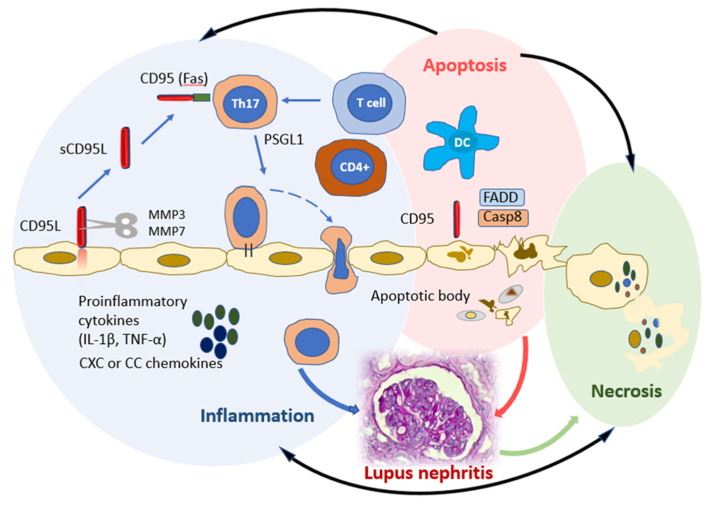
CD95 stimulation and potential links to inflammation activation. Three molecular complexes can occur with CD95 involvement to induce chronic inflammation (blue circle), apoptosis (pink circle), or necrosis (green circle). Apoptotic and necroptotic complexes control each other and the inflammatory complex (black lines). CD95 (FAS) promotes DC maturation and differentiation of Th17 and induction of the secretion of pro-inflammatory cytokines (IL-1β, TNF-α) and CXC or CC chemokines. The CD95-mediated apoptotic and non-apoptotic signaling pathways share many factors, such as the apoptotic factors FADD and caspase-8. Interaction of CD95 L with CD95 favors the recruitment of FADD to the death domain of CD95. Abbreviations: Th, T helper; DCs, dendritic cells; IL, Interleukin; TNF, Tumoral necrosis factor; CD95L, CD95 Ligand; MMP, Matrix metalloproteinase; PSGL-1, P-selectin glycoprotein ligand-1; FADD, Fas-associated death domain; Casp8, Caspase-8.

More recent data demonstrate that CD95 is involved not only in the process of apoptosis, but also in the development of inflammatory processes [21]. CD95 stimulation and the potential associations with inflammation activation are presented in Figure 1.

The activation of CD95 on immature DCs causes their maturation and induces the secretion of proinflammatory cytokines IL-1β, TNF-alpha (α), and CXC or CC chemokines, which may play important roles in the recruitment, activation, and proliferation of naïve T cells [21]. On the other hand, the stimulation of the recognizing receptors on DCs increases CD95 expression and mediates their destruction by activated T lymphocytes expressing CD95L. CD95 knockout on DCs and CD95L on T and B lymphocytes cause autoimmune processes due to the resistance of such cells to CD95L-mediated apoptosis and the accumulation of DCs in the focus of the inflammation [22,23]. CD95 expression on CD4+ lymphocytes is necessary for the differentiation of type 17 (Th17) T helper cells. Th17 cells play one of the leading roles in the development of inflammatory and autoimmune processes [24]. The loss of CD95 leads to a loss of T-cell polarization in Th17, which is better activated in the presence of TGF-β and IL-6. It has been shown that the loss of CD95 contributes to Th1 differentiation compared to Th17 differentiation [25]. CD95 mutations are also associated with deregulated B-lymphocyte function, which predisposes patients to systemic autoimmunity, including SLE. A unique subpopulation of B cells with CD27-Syk++CD38-CD95+ immunophenotype and characterized by increased phosphorylation of Syk tyrosine kinase was found in patients with SLE, which may also indicate the role of CD95 in the pathogenesis of ARDs [26]. Some studies have demonstrated the importance of CD95 expression in assessing SLE activity. So, D. Adlowitz et al. [16] found that CD95 expression reflects the increased activation of B lymphocytes and a good response to anti-B-cell therapy. In the study of A. Jacobi et al. [27], it was shown that an increased content of CD27-IgD-CD95+ B lymphocytes in the blood is detected during an SLE flare, and the absolute number of these cells correlates with the activity of the disease.

Fas is important in antibody affinity maturation. In germinal centers, Fas and CD40 signaling are counteracted to each other. Fas signaling can prevent lymphoproliferative disorders by the elimination of self-reactive antibodies. In contrast, CD40 co-stimulation promotes maturation of the antibody response by participating in the selective rescue of B-cell clones with high affinity antigen receptors from Fas-mediated deletion [28]. An experimental model has been demonstrated that explains the inverse correlation between antibody affinity and activity, which may be relevant to future drug discovery efforts on targets in the TNF receptor superfamily [29].

Therefore, continued investigation into the biochemical, cellular, and clinical contexts of the CD95 pathway in the broad spectrum of inflammatory diseases will hopefully continue to provide insights into both the pathogenic relevance and the therapeutic capabilities of CD95 pathway modulation.

### 3.2. HLA-DR

HLA-DR is a class II MHC cell surface receptor; it is a ligand for TCR. The autoimmune disorders in ARDs are associated with a higher level of T-cell activation, which is apparently associated with the abnormal signaling of TCR [30]. To date, the expression of HLA-DR on CD27++CD20-CD19dim B cells has been characterized only in a small number of SLE patients as most studies focus on their characterization as immunoglobulin (Ig)-producing cells. However, it seems to be extremely important in patients with ARDs to analyze the ratio of HLA-DRhigh/HLA-DRlow cells in order to differentiate newly generated plasmablasts from mature plasma cells. A. Jacobi et al. [17] observed an increased number of circulating CD27highHLA-DRhigh cells in SLE patients compared with the healthy controls. The results also indicate that newly differentiated HLA-DRhigh plasmablasts predominate over HLA-DRlow plasma cells in the peripheral blood of SLE patients, and this balance depends on SLE activity. HLA-DRhigh plasmablasts are also found in the bone marrow aspirates of SLE patients. This population is a more accurate marker of B-cell activation in SLE, which is closely related to the activity of the disease, including the production of IgG Abs against double-stranded DNA (anti-dsDNA). It is possible that the expansion of HLA-DRhigh plasmablasts in SLE is the result of the premature activation and differentiation of B cells. This assumption is confirmed by the fact that the content of CD27+IgD+CD95+ memory B cells with an activated phenotype is increased in patients with active SLE [27]. However, whether they transform themselves or only replace the preceding plasma cells and undergo apoptosis in individual patients remains to be studied.

In addition, the increased expression of HLA-DR+ was demonstrated in both the CD4+ and the CD8+ T-cell subpopulations of SLE patients compared to the healthy donors. Earlier studies showed that the frequency of CD8+HLA-DR+ T cells strongly correlates with SLE flare [31]. It turned out that the HLA-DR molecule was expressed on the circulating CD4+ T cells of patients in the range from 1 to 24%, and the percentage of cells with this late activation marker depended on the activity of the disease determined by the Systemic Lupus Activity Measure (SLAM) [32]. Elevated frequencies of HLA-DR+ T cells were observed in SLE patients with positive anti-dsDNA compared with the healthy controls. The expression of HLA-DR+ T cells was positively correlated with the Systemic Lupus Erythematosus Disease Activity Index (SLEDAI) [33]. The study of the expression of HLA-DR may be of clinical importance for monitoring exacerbations and remissions during the course of SLE.

Analysis of the co-expression of CD38 and HLA-DR activation molecules on T lymphocytes revealed a higher frequency of CD38+HLA-DR+ in CD4+, CD8+, and CD8+ Temra memory cells (memory T cells that re-express CD45RA) in SLE patients than in individuals without ARDs [34]. In the subpopulations of CD4+ effector cells of patients with SLE, a higher content of activated Th1 lymphocytes (8%) was found compared to the control group (4.3%, *p* < 0.05). The association of CD38+HLA-DR+ expression on activated CD4+, Th1, and Tfh memory T cells with B-cell switching and plasmablast differentiation in patients with SLE has been demonstrated. Thus, a higher frequency of subpopulations of HLA-DR+CD38+ blood T cells and their association with subtypes of B lymphocytes are presented as key differences between patients with SLE and healthy donors.

### 3.3. CD25

CD25 is a protein from the group of leukocyte differentiation antigens and is an α chain of the IL-2 receptor (IL-2Ra). CD25 is expressed in developing and activated T lymphocytes, activated B lymphocytes, myeloid cell precursors, and oligodendrocytes. 

With the accumulation of data on the involvement of the T-cell component in the pathogenesis of autoimmune diseases, interest in the markers of T-lymphocyte activation, including CD25, grew. In some studies, a lower content of CD4+CD25+ T cells was determined in the peripheral blood of SLE patients than in the control group [35,36]. In a study by A. Sonawale et al. [37], an association was found between the expression of CD4+ and CD25+ molecules with the SLEDAI.

The determination of the CD25 molecule is also critically necessary when analyzing the expression of the Treg immunophenotype because the assessment of ICP expression in this subpopulation is crucial to establishing its functional activity and role in the pathogenesis of ARDs. Some of the studies have found a direct relationship between the number of CD25+ and a decrease in the number of Tregs (CD4+CD127-/lowCD25+). An association of the number and the functional status of Tregs with the activity and prognosis in SLE has been demonstrated [38]. Thus, the CD4+CD25+ population can be considered as a sensitive specific marker of SLE activity.

## 4. Immune Checkpoints and Autoimmune Rheumatic Diseases

In recent years, a lot of information has appeared about the effect of various molecular mechanisms, called immune checkpoints (ICPs), on immunological effector cells. ICPs are receptors on the surface of T lymphocytes and other cells. These receptors bind to the corresponding ligands that are located on the cells and suppress the T-cell immune response.

ICPs are a system of inhibitory/activation mechanisms that regulate the development of the immune response, including preventing the launch of autoimmune processes, as well as modulating them, reducing the damage caused by immune cells in organs and tissues. Normally, ICPs serve to prevent autoimmune tissue damage [39]. At the moment, more than twenty such molecules are known, and their list is constantly being updated. 

The use of ICP inhibitors in the treatment of cancer has demonstrated the development of adverse immunological reactions. This fact allows the discussion of the commonality of malignant and immunological disorders [40]. 

The roles of ICPs in maintaining immune tolerance and hence suppressing autoimmunity were revealed in animal models and validated by the clinical successes of ICP-targeted therapeutics for autoimmune diseases [41]. In addition to the promising application of immunotherapeutic blocking and activating drugs to these molecules, studies indicate their connection with the pathogenesis and degree of activity of ARDs [19,20,42,43,44]. In the light of the ongoing development of new drugs aimed at ICPs for the treatment of autoimmune and oncological diseases, the determination of ICPs is critically necessary for the personalization of such therapy and the correct assessment of its effectiveness. Table 1 presents the most significant ICPs for the development of ADRs and their effects on immune system cells, which will be discussed later. 

## 5. Immune Checkpoints Are Promising Targets for Targeted Immunotherapy of Autoimmune Rheumatic Diseases

Molecules capable of regulating the activation of the immune response are a promising target for the use of immunotherapeutic drugs in ARDs. It seems that for ARDs the most important and perspective ICPs are OX40, GITR, CD278 (ICOS)PD-1, CTLA-4, and CD40, which modulate the immune response towards activation or suppression [23]. 

### 5.1. OX40

**Figure 2:**
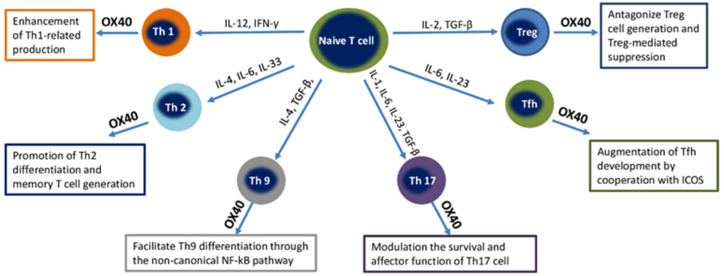
Effect of the OX40-OX40L interaction on different T-cell subsets. Abbreviations: Th, T helper; IL, Interleukin; IFN-γ, Interferon gamma; TNF, Tumoral necrosis factor; TGF-β, Transforming growth factor beta; Tregs, T regulatory cells; Tfh, T follicular helper cells; NF-κB, nuclear factor kappa-light-chain-enhancer of activated B cells.

OX40 (CD134), the molecule, also called CD134, uses OX40L or CD252 as its ligand. OX40 is a membrane-bound glycoprotein of the TNF superfamily, which is mainly found on activated CD4+ T cells. The OX40 ligand (OX40L) is expressed on activated APCs, including B cells, macrophages, endothelial cells, and DCs [66]. OX40 expression is absent on human naive CD4+ T cells, and the constitutive expression of OX40 is reported in mouse Tregs. OX40 expression is observed on activated memory cells and regulatory CD4+ T cells; less OX40 expression was found on activated CD8+ cells. OX40 expression is considered as a marker of thymic T cells receiving positive selection signals. In addition, a lower level of OX40 expression is observed on the T cells of natural killer T cells (NKT cells), natural killer cells (NK cells), and neutrophils [67]. The effect of the OX40–OX40L interaction on T-cell subsets is illustrated in Figure 2. 

OX40–OX40L signals can enhance the Th1-mediated immune response, promote generation of Th2, favor Th9 differentiation through the non-canonical NF-κB pathway, augment Tfh development, and antagonize Treg generation and Treg-mediated suppression.

The interaction of OX40 with activated T lymphocytes during the antigen-specific stimulation of T cells can save effector T cells from peripheral deletion. In a study by R. Kumari et al., a higher expression of OX40 on T-effector (CD4+Foxp3-) and Treg-Treg (CD4+Foxp3+) was found in the bronchoalveolar lavage fluid compared with the peripheral blood of patients with pulmonary sarcoidosis [68]. However, OX40 exerted a contrasting impact on these T-cell subsets, enhancing effector T-cell functions (INF γ, TNF-α), while inhibiting Treg function (IL-10, TGF-β). 

This leads to an increase in the survival of T cells during the primary immune response and their transition to memory T cells, which, in the case of interaction with a specific antigen, can lead to the development of an autoimmune disease [69]. In addition, OX40L enhances the proliferation and differentiation of B cells. This mechanism causes B-cell hyperactivity, detected in SLE. OX40L negatively regulates the generation and function of IL-10-producing Tregs, which play a critical role in maintaining peripheral tolerance [70]. The TNFSF4 gene carriage is associated with increased OX40 expression [71]. It is assumed that the increased expression of the TNFSF4 gene predisposes to SLE either by quantitatively increasing the interaction of T lymphocytes with APC or by influencing the activation products of Treg-Treg.

The number of peripheral CD4+ T cells expressing OX40 is usually increased in patients with ARDs. A number of studies indicate a correlation between the expression of OX40 and the severity of SLE [45]. The therapy for other autoimmune diseases may reduce the proportion of circulating lymphocytes expressing OX40. Thus, the use of natalizumab in multiple sclerosis leads to a decrease in the expression of OX40 on the surface of CD4+ T cells [46]. Similarly, in patients with type 1 diabetes mellitus (DM), as well as prediabetic probands, the co-expression of OX40 and CD25 (IL2Ra) is strongly associated with the identification of autoreactive T cells, which may confirm their role in the initiation of the disease [26].

When analyzing the presence of OX40 on lymphocytes, it was found that the content of CD4+ T lymphocytes expressing the OX40 molecule was significantly higher in patients with SLE than in the control. OX40 expression was highest in patients with lupus nephritis, especially class V, and positively correlated with blood creatinine and SLEDAI-2K. The expression of OX40 on CD4+ T cells had a higher significance and specificity in the diagnosis of lupus nephritis than the levels of OX40L and dsDNA Abs [24].

Antigen-presenting cells from patients with active SLE mediated Treg dysfunction in an OX40L-dependent manner, and the OX40L-expressing cells colocalized with the Foxp3+ cells in active SLE skin lesions. Engagement of the OX40L/OX40 axis resulted in Foxp3 downregulation in Tregs, and the expression in SLE Tregs correlated with the proportion of circulating OX40L-expressing myeloid DCs [47].

All of the above facts make OX40 a promising target for the use of immunotherapeutic drugs. The blockade of the OX40–OX40L interaction can lead to the inhibition of proinflammatory cytokine production in vitro and to the suppression of the progression of collagen-induced arthritis (CIA) in DBA/1 mice [48].

A preliminary clinical study shows that therapy neutralizing IL-17 due to the OX40–OX40L blockade can suppress inflammatory cascades in peripheral joint synovium in patients with active RA [72].

In a randomized, placebo-controlled clinical study, oxelumab, an anti-OX40L antibody, was examined in a patient with mild asthma, but it did not provide a benefit to patients [49]. Administration of ligand-blocking anti-mouse surrogate Abs OX40.23 or BMS-986178, alone or in combination with checkpoint blockade, resulted in increased activation of peripheral CD4+ and CD8+ T cells in mice with tumors and in patients with solid tumors. A study of another anti-OX40 antibody [50], GBR 830, showed significant clinical improvement in patients with moderate to severe atopic dermatitis, together with decreases from the baseline in OX40-expressing T cell and OX40L-expressing dendritic cells in lesional skin.

Another OX40L antibody (KY1005) examined in atopic dermatitis patients was engineered to be non-depleting, which was prudent given that this receptor is expressed in an inducible manner on a variety of cells, but the results have not been posted yet [NCT03754309].

However, when evaluating the results of the study, it should be remembered that the expression of OX40 is inducible and dynamically changes. This significantly complicates the selection of the doses and treatment regimen with such drugs. Thus, it is necessary to correctly select the timing and evaluate the expression of the checkpoint on the cell surface before prescribing the drug.

### 5.2. GITR 

The activation of another promising target of immunotherapy—the GITR molecule, which triggers and enhances the immune response, is also indicated in autoimmune diseases. Glucocorticoid-induced TNFR family-related receptor (GITR) is a surface receptor molecule that is involved in inhibiting the suppressive activity of Tregs and in prolonging the survival of T effector cells [51].

On naive T cells, GITR is expressed to a low degree, with a significant increase after activation of TCR [73]. With a high degree of expression, GITR is represented on CD4+CD25+FoxP3+ Tregs. The GITR molecule is also present on the membrane of NK cells and, to a lesser extent, B lymphocytes, macrophages, and DCs [74]. GITR stimulation has an activating effect on ordinary T cells, increasing their survival, activation, and proliferation by increasing the TCR-induced proliferation, cytokine production, and blocking anti-CD3-induced apoptosis of T cells [75]. Further studies indicate a different role of GITR in CD4+ and CD8+ T cells. It has been shown that GITR can lower the threshold of CD28 co-stimulation in effector CD8+ T cells. GITR activity on CD8+ T cells is considered to be independent of CD28 activation, unlike that of CD4+ T cells [76]. In effector CD4+ T cells, GITR stimulation on CD4+CD25-T cells can induce the survival, activation, and proliferation of CD4+ T cells, and this effect, conversely, mainly depends on the joint stimulation of TCR and CD28 [77].

In recent years, the regulation of the interaction of GITR and its GITRL ligand has been studied in animal models of autoimmune diseases. Thus, the introduction of recombinant GITRL to mice with CIA contributed to an earlier onset and severe course of the disease, accompanied by the accumulation of Th17 cells in the spleen and lymph nodes. In vitro studies have shown that GITRL can effectively promote the differentiation of naive CD4+ T cells into Th17 cells [78]. As is well known, Th17 cells play an essential role in the pathogenesis of RA. Further studies have shown that GITRL initiates the p38 MAP signaling pathway and activates STAT3 signaling, which is responsible for the development of Th17 cells [79]. In addition, the splenic Tfh cells of mice with CIA were found to express higher levels of GITR, and GITR activation significantly increases the percentage and the number of Tfh cells in vitro and in vivo. Blocking GITR/GITRL interaction by the GITR-Fc protein reduced the severity of the disease by suppressing the cellular response of Tfh [80]. Taken together, all these studies point to the critical role of GITR/GITRL signaling in the CIA mouse model.

The studies revealed an increase in the level of GITR in the blood serum and synovial fluid in RA patients compared with those without ARDs. Positive correlations were found between the level of GITRL in the blood serum and the parameters of inflammation (C-reactive protein (CRP), the erythrocyte sedimentation rate, and the production of autoAbs (IgM and IgG rheumatoid factor (RF)), which may indicate the role of GITRL in the development of RA [81]. 

Immunohistochemical analysis of synovial fluid revealed the increased expression of GITR in macrophages in RA in contrast to osteoarthritis. The stimulation of macrophages with monoclonal Abs (mAbs) against GITR in RA induced the regulation of the intercellular adhesion molecule (ICAM) and the expression of pro-inflammatory cytokines/chemokines and matrix metalloproteinase-9 in synovial macrophages [52]. These data indicate that GITR can enhance the inflammatory activation of macrophages by promoting cytokine gene expression and adhesion between the cells and the extracellular matrix in the synovial membrane in RA; thus, macrophages function as pro-inflammatory agents in the development of ARDs in a GITR-dependent way.

The overexpression of GITR was found in subpopulations of Tregs and CD4+CD25- in SLE patients compared with healthy donors. Patients with active SLE had higher GITR expression on CD4+CD25+, CD4+CD25high, and Tregs, but not on CD4+CD25- cells, compared with patients with remission/low SLE activity. GITR expression levels on CD4+CD25+, CD4+CD25high, Tregs, and CD4+CD25- cells positively correlated with the SLEDAI scale. A negative correlation of GITR expression on CD4+CD25- and Tregs with the level of C3 in the blood of SLE patients was established. No correlation was found between the expression of GITR and the levels of ESR, CRP, C4, and autoAbs (anti-C1q, ANA, anti-dsDNA, anti-Sm, anti-SS-A, anti-SS-B, anti-RNP, and anti-nucleosomal Abs) [53]. It has also been demonstrated that the content of CD4+CD25low/-GITR+ lymphocytes is elevated in peripheral blood in 50% of SLE patients. Phenotyping of lymphocyte subpopulations showed that in SLE patients CD4+CD25low/-GITR+ cells are fully active Tregs, possibly representing peripheral Tregs [54]. Their content increases in patients with inactive disease. These data may indicate the key role of this subpopulation of T cells in the modulation of an abnormal immune response in SLE [55].

Thus, it is likely that the blockade of GITR, which leads to the inhibition of the activation of autoreactive T lymphocytes and to maintaining the immunocompetence of Tregs and myeloid suppressor cells, may be promising for suppressing the excessive autoimmune process. To date, several studies have confirmed the therapeutic effects of GITR-Fc protein fusion or GITR gene knockout in mouse models of autoimmune diseases [56]. However, before the start of clinical trials on patients, a more detailed study of ICs and GITR in the pathogenesis of ARDs is required.

The inhibition of GITR triggering by GITR-Fc fusion proteins plays an anti-inflammatory role in several murine models of autoimmune/inflammatory disease. However, several points are still unclear. Unraveling the function of GITR and its ligand in specific subsets of immune cells will allow for the development of pharmacological tools that are more active [77]. 

Consistent with the co-activating effect of agonist mAbs on conventional T cells, which results in increased anti-tumor effects and infection resistance, the decrease in inflammatory and autoimmune diseases in GITR−/− mice also indicates a possible therapeutic effect of GITR antagonism. In this respect, antagonist Abs and fusion proteins should be considered in the future for the establishment of anti-inflammatory therapy. Notably, in the case of inflammatory/autoimmune diseases, the agonist/antagonist activity of therapeutic agents should be determined before clinical use. Moreover, the possibility of using Abs with specific stimulating effects on Tregs that promote their expansion and long-term survival and result in immunosuppressive/anti-inflammatory activity should be evaluated for therapeutic use [82].

Moreover, some effects were observed with the use of GITR agonists to activate Treg effects [57]. Thus, different effects of GITR stimulation (activation of effectors or Tregs) can be used to reduce the immune response in ARDs, and the choice of a specific mechanism apparently depends on the specific state of the immune system.

### 5.3. ICOS

Inducible T-cell co-stimulator (ICOS) is a homodimeric protein with a molecular weight of approximately 55~60 kD that was initially identified on the surface of T cells during TCR stimulation [58]. The gene encoding ICOS is in close proximity to the genes encoding CD28 and CTLA-4 [83]. In this regard, ICOS has significant homology with other ICPs, the co-stimulatory molecules CD28 and CTLA-4. 

ICOS is not expressed on resting T cells. ICOS expression requires signaling via TCR and/or CD28 co-stimulation. ICOS is expressed on activated CD4+ and CD8+ T cells along with CD28 and CTLA-4. This suggests that ICOS regulates the adaptive T-cell response similarly to the CD28 and CTLA-4 activity [84,85]. A study of ICOS distribution on T cells found its expression on Th1, Th2, Th17, Tfh, T follicular regulatory cells (Tfr), Tregs, type 1 regulatory T cells (Tr1), and innate lymphoid cells, as well as a small CD3+ subpopulation of T cells [86,87]. In addition, ICOS has a unique ligand, ICOSL, which is expressed on the surface of almost all APCs, confirming the leading role of ICOS in immune responses.

According to several studies, ICOS is involved in the differentiation, proliferation, and survival of Tregs, providing them with immunosuppressive capacity. ICOS can mediate the generation of FOXP3+ Tregs. They have a high level of constitutive ICOS expression, which suggests the involvement of ICOS in the functional activity of Tregs [88].

Homozygous deletion of the ICOS gene in patients with common variable immunodeficiency (CVID) leads to suppression of Treg induction and loss of auto-tolerance [89]. Q. Chen et al. showed in a male BALB/c mouse model that the Treg-associated exit from the immune response may be mediated by ICOS signaling, which facilitates NFAT:Foxp3 interaction in favor of suppressive Treg function [90]. These phenomena confirm the importance of ICOS for the transcriptional activity of FOXP3+. 

The ICOS signal promotes Treg proliferation. The proliferative ability of ICOS+ Tregs was confirmed by higher Ki-67 expression on these cells [91]. ICOSL expressed on plasmacytoid DCs preferentially promotes ICOS+ Treg proliferation by interacting with ICOS on Tregs in vitro [92]. ICOS expression indicates a stronger suppressive immunosuppressive activity of Tregs [93]. Similarly, ICOS+ Tregs have been proposed as a dominant subset of Tregs to prevent the development of DM in non-obese diabetic (NOD) mice. A dramatic decrease in ICOS expression on pancreatic Tregs was observed as the mice developed DM [91].

In vivo experiments have shown that the ICOS plays an important role in the regulation of the humoral immune response, namely in the formation of germinal centers and the generation of Ig class switching [94]. Long-lived plasma cells and memory B cells that have undergone class switching and somatic hypermutation to increase antibody affinity are products of germinal center reactions. These cell types and the Abs they produce are considered absolutely critical in the maintaining of the protection against pathogens or in the development of a number of autoimmune diseases [84].

It has been observed that ICOS expression is increased in a number of autoimmune diseases and various types of neoplasms. Thus, the progression of type 1 DM was accompanied by a decrease in ICOS expression by islet Tregs. ICOS+ Tregs, in contrast to ICOS- Tregs, have a higher ability to proliferate and suppress in situ IL-10 secretion. An ICOS deficiency or blockade impaired the competitive fitness of the Tregs and did not protect NOD mice from developing type 1 DM [91]. 

In SLE, increased levels of circulating CD4+ ICOS+ Foxp3+ T cells contribute to IL-10 production and correlate with disease severity, the SLE activity index, and serum anti-dsDNA (although the authors suggested that these ICOS+ Tregs may be the precursors of inflammatory cells) [95].

The impaired differentiation of ICOS+ Tregs is likely to be involved in the transition from low to high disease activity in SLE. In SLE patients in remission, ICOS differentiation was observed for all subsets of Treg/responder T cells (Tresps). Increased conversion of mature naive Treg/Tresps at rest specifically warranted a significantly increased ratio of ICOS+-Treg/ICOS+-Tresps and ICOS--Treg/ICOS-Tresps. In the case of SLE flares, the change in resting Tresp differentiation caused a significant change in the ICOS+-Treg/ICOS+-Tresp ratio in favor of ICOS+-Tresps. Thus, ICOS+- recent-thymic-emigrant (RTE)Treg/Tresp differentiation appears to play a crucial role in maintaining SLE remission, and the production of proliferating resting mature naive (MN)Tregs may be responsible for active disease [96]. P. Shaktivel et al. [97] reported a high level of ICOS expression in pulmonary Tregs and its association with the prognosis in patients with pulmonary sarcoidosis. 

In addition, the molecule may be a predictor of treatment response as a significantly higher proportion of ICOS+ Tregs was found in RA patients who did not respond to methotrexate therapy [98]. AMG 557 (also known as prezalumab), a human IgG2 mAb that binds with high affinity to ICOSL and prevents its interaction with ICOS, was developed. A significant decrease in IgG against neoantigen (which was hemocyanin of molluscum) was observed in SLE patients receiving AMG 557 injections [99]. In clinical trials, the using of this drug showed the safety of its administration. The clinical outcome was different across trials and diagnoses: in SLE patients (NCT02391259 and NCT00774943), there was no significant clinical effect, but in an NCT01683695 SLE study, an improvement of SLEDAI was observed [100]. 

In patients with active primary-Sjogrens-syndrome, treatment with MEDI5872 did not result in a consistent improvement in clinical or other biomarker measures of disease activity, despite a decrease in RF levels [101]. Similar to the above antibody, MEDI-570 is an afucosylated human IgG1κ mAbs, directed against the ligand binding domain of human ICOS. MEDI-570 displays relatively high (picomolar) binding to ICOS, which abrogates ICOS-mediated T-cell proliferation and cytokine production. A phase I study to evaluate the safety and tolerability of MEDI-570 was initiated in SLE (NCT01127321). Enrolment into higher doses was stopped even though dose-limiting toxicities were not identified. Obviously, it is necessary to continue research for a set of representative samples and the selection of the optimal administration mode.

Thus, based on the data on the functional activity of the molecule, ICOS can be considered as a potential biomarker of ARDs activity and prognosis, and the effectiveness of various treatment regimens.

### 5.4. PD-1

Programmed cell death protein 1 (PD-1), also known as CD279, is a transmembrane protein involved in the immune system suppression. PD-1 expression on T/B cells regulates peripheral tolerance and autoimmunity [59]. The binding of PD-1 to its ligand, PD-L1, plays an important function in immune tolerance [60]. PD-1 protects against autoimmunity through two mechanisms. First, it promotes apoptosis of antigen-specific T cells in the lymph nodes, and second, it reduces apoptosis in Tregs [60,61]. 

PD-1 inhibits T-cell recruitment into the follicle by the suppression of phosphoinositide-3 kinase (PI3K) activities downstream of the follicle-guidance receptor CXCR5; it is independent of the co-signaling with the TCR and necessitates the overcoming of the ICOS signaling. PD-1 expression causes a concentration of Tfh cells toward the germinal center; the territory is restricted by their CXCR3 upregulation. In that zone, the PD-L1-PD-1 interactions between the individual Tfh and the B cells optimized the B-cell competition and affinity maturation. Therefore, PD-1 controls the tissue positioning and function of the Tfh cells. In contrast, ICOS activates T-cell persistent motility at the T–B border and their recruitment to follicles. Moreover, ICOS co-stimulates the calcium fluxes in the Tfh cells during the antigen-specific interactions with GC B cells and causes the selection of high-affinity B cells through the ICOS-CD40 intercellular positive signaling between the Tfh and the B cells [102].

In fact, PD-1 deficiency leads to spontaneous autoimmunity in mouse models of type 1 DM and SLE [103]. Studies have shown that PD-1 gene knockout (Pdcd1−/−) mice develop lupus-like arthritis, glomerulonephritis, and autoimmune dilated cardiomyopathy in BALB/c mice [104,105]. In humans, polymorphism of the gene encoding PD-1 has been significantly associated with a higher prevalence of RA and SLE [106,107]. 

A number of RA studies have reported PD-1 expression on T cells infiltrating the synovial membrane, synovial fluid, and peripheral blood, as well as the PD-L1 expression on the synovial tissue and synovial fluid [108,109,110]. Higher PD-L1 expression on the synovial lining cells was associated with an RF positive state, more infiltrating CD3-positive T cells, higher CRP, and more pronounced synovitis in RA [109].

The results of Y. Huang et al. showed that the expression levels of PD-1 and OX40 in CD4+ T lymphocytes were markedly increased in RA patients and CIA mice [111]. The authors further found that decreased PD-1 expression increased the serum levels of IgG, IgG1, and IgG2a in CIA mice and also increased the levels of IL-4, IL-2, IL-5, IL-17, and IFN-γ in the spleen cells and articular tissues of mice.

The expansion of B cells in SLE [112] may suggest that B-cell PD-1 is not effectively expressed or ligated in SLE despite increases in the transcript level. PD-L1 expression is upregulated on SLE patient peripheral blood neutrophils [113] but reduced on DCs and monocytes [114]. Possibly, macrophages in SLE also express PD-1 as a biomarker of their reduced ability to clear apoptotic cells [115]. Anti-PD-1 antibodies are elevated and positively associated with the SLEDAI score [116]. The production of PD-1 antibodies in SLE may break the immune tolerance established by the PD-L1 expression on epithelial and endothelial cells [117], resulting in nephritis, similar to the cases identified with PD-1 therapies [118].

Several studies have shown that PDL1 expression on nonhematopoietic cells may suppress the infiltration of autoreactive T cells into target organs and inhibit immune-mediated tissue damage caused by the response of autoreactive effector cells. Thus, deficiency of the PD-1 pathway may enhance autoimmunity, leading to dacryoadenitis in mice [119]. In Sjögren’s syndrome, PD-L1 expression was observed on the ductal and acinar epithelial cells of the salivary glands, which correlated with the degree of lymphocyte infiltration [120]. Dysregulation of the immune system through the PD-1/PD/L1 pathway may be primarily responsible for the more frequent cases of malignization in ARDs. 

Agonists against PD1 are at an early stage of clinical development and include the antibody CC-90006, which is currently in phase I trials for psoriasis (NCT03337022). 

### 5.5. CTLA-4

CTLA-4, a leukocyte differentiation antigen (CD152) and a transmembrane receptor on T cells, plays an important role in the negative regulation of T-cell response [62]. CTLA-4 is a highly endocytic molecule that binds to two different ligands, CD80 and CD86, resulting in their removal from opposing cells. Using transendocytosis, CTLA-4 can act as an immunoregulatory mechanism, directly reducing the ability of APCs to stimulate via CD28. This concept helps to explain why the stimulatory and inhibitory receptors share the same ligands and encompass the endocytic nature of CTLA-4. Taken together, this model suggests that the CD28/CTLA-4 system functions as a rheostat that can increase or decrease T-cell activation [121]. Anti-CTLA-4 Abs bind to CTLA-4 molecules with high affinity, resulting in Treg depletion or functional blockade, leading to increased T-cell activation and immunological response. The effects of the CTLA-4 blockade may be mediated by various mechanisms: prevention of transendocytosis, increased levels of CD80/CD86 on APC, direct Treg cytotoxicity, and antibody-dependent cellular cytotoxicity mediated by FcR-IV-expressing macrophages. TLA-4-deficient mice suffer an autoimmune disease characterized by polyclonal T-cell proliferation, which supports a critical role for CTLA-4 in controlling T-cell responses [122]. In a recent study by L. Zhao et al. [123], it was shown that the CTLA-4 expression was reduced during induced CD4+CD25+Foxp3+ Tregs in SLE patients, which correlated with the SLE activity.

Further evidence for a role of CTLA-4 in the control of polyclonal autoreactive T cells has come from examining the circulating TCR repertoire following anti-CTLA-4 therapy [124]. A large number of studies in animal models, as well as the results of clinical trials, demonstrate the safety and efficacy of CTLA-4-Ig for the inhibition of T-cell responses in the immunotherapy of ARDs. Abatacept with the CTLA4-Ig fusion protein shows efficacy in the treatment of autoimmune disorders, including RA [125]. In patients with Sjögren’s disease, treatment with abatacept reduces the amount and percentage of circulating Tfh and the expression of the activation marker ICOS on CD4+ T cells [126]. The therapeutic potential of CTLA-4-Ig might also augment in combination with PD-1:PD-L modulation [127].

### 5.6. CD40

CD40 and CD40 ligand (CD40L) are stimulatory immune checkpoints that play a broad role in various immunological processes. CD40 belongs to the TNF receptor superfamily that is expressed mainly on B cells. It was described as a regulator of B-cell proliferation [63]. To date, it has been shown by RNA sequencing that CD40L is mainly expressed in T cells [64]. The biological function of the CD40/CD40L interaction plays a crucial role in the humoral immune response by T-cell-dependent B-cell differentiation and activation and in mediating a bi-directional dialogue between T cells and APCs, which provides an amplification loop in cellular immunity [65]. CD40 stimulation results in the upregulation of CD80, CD86, CD95/Fas, and MHC class II on human B cells, and the production of CD40 co-stimulates the production of pro-inflammatory cytokines, including IL-12, IL-6, TNFα, and lymphotoxin-alpha. In the presence of IL-4 signaling, CD40 promotes secretion of IgM, IgG, and IgE antibodies, while CD40 stimulation in the presence of IL-10 and TGF-β can induce IgA secretion. In combination with IL-21, CD40 induces the differentiation of naïve and memory human B cells into CD38hi plasma cells and induces a class switch recombination to IgG1 and IgG3 from naïve B cells. Due to the activation role of this interaction, it is logical to assume the central part played by these molecules in ARD development. Overexpression of CD40L was described on B and T cells in patients suffering from SLE, RA, and psoriatic arthritis [128], while the level of its expression on T cells correlated with poor outcome, disease severity, and flares [129].

An important place for CD40 expression in the development of ARDs was described for RA, Sjögren’s disease, multiple sclerosis, and other ARDs [130,131,132].

Based on these data, attempts were launched to create therapeutic antibodies capable of blocking the СD40-CD40L interaction and reducing the pathological activation of the immune system. One of the first drugs was anti-CD40L mAb (IDEC-131). Despite the fact that some studies have shown significant improvement in several disease activity parameters (such as the production of anti-dsDNA, hematuria, and the concentration of complement 3 and renal function), increased incidence of thromboembolic events due to crosslinking CD154 expressed on platelets was observed [133]. To resolve this problem, new anti-CD40L drugs, dapirolizumab pegol and VIB4920, were designed and examined in the first stages of clinical trials. The study showed the safety of their administration and the reduction in disease activity [134,135]. For the blocking of CD40, two mAbs were designed. First, CFZ533 was examined on Sjörgen syndrome and RA patients. These trials showed the safety of CFZ533 administration for both diseases and a significant improvement of the clinical parameters in Sjörgen syndrome [136,137]. BI 655064, another anti-CD40 mAb, also did not show adverse events and was associated with a reduction in activated B cells, autoantibody production, and inflammatory and bone resorption markers, but significant effects on clinical outcome were not observed [138].

Thus, the blocking of the CD40–CD40L (CD154) costimulatory pathway is a promising method in the treatment of ARDs, but the trials require a larger number of patients.

## 6. Conclusions

The expression of activation markers on B and T cells, such as CD95, HLA-DR, and CD25, has been shown to correlate with the degree of ARD activity and to have prognostic value in assessing the effectiveness of therapy 

ICPs, such as OX40, GITR, ICOS, PD-1, CTLA-4, and CD-40 are currently being considered as promising targets for targeted immunotherapy for ARDs. It seems promising to link the evaluation of B- and T-cell populations, their activation profile, and the expression of ICPs; the goal is to find out the complex mechanism of their interaction and the effect of ICPs on various immune and non-immune cells contributing to the initiation and progression of ARDs.

In light of the ongoing drug development and trials, the study of ICP activation/blocking is essential to personalize therapy and provide more effective treatment for ARDs [139].

## Figures and Tables

**Table 1:** Immune checkpoints expression and functions/properties.

Immune Checkpoints	Expression and Functions/Properties	References
OX40 (CD134)	Mainly expressed on activated APCs	[34]
	Expressed on hematopoietic cells such as activated NK cells, NKT cells, mast cells or the responding CD4+ T cells, and non-hematopoietic cells, such as endothelial cells or smooth muscle cells	[35,36]
	An increase in the survival of T cells and their transition to memory T cells during the immune response	[37]
GITR	Inhibition of suppressive activity of Tregs and prolongation of T-effector-cell survival	[45,46]
	Present on the membrane of NK cells and, to a lesser extent, B lymphocytes, macrophages and DCs	[47]
	Increases survival, activation, and stimulation of T-cell proliferation by increasing TCR-induced proliferation, cytokine production, and blocking anti-CD3-induced T-cell apoptosis	[48,49]
	Differentiation of naive CD4+ T cells into Th17 cells	[50]
	Activation of the p38 MAP signaling pathway and the STAT3 signaling pathway influencing the development of Th17 cells	[51]
ICOS	Expressed on activated CD4+ and CD8+ T cells, presumably regulates adaptive T-cell response	[52,53]
	Expressed on Th1, Th2, Th17, Tfh, Tfr, Treg, Tr1, and innate lymphoid cells	[54,55]
	Participation in differentiation, proliferation, and survival of Tregs	[56,57,58]
	Regulation of the humoral immune response, namely in the formation of germinal centers and the generation of Ig class switching	[55]
PD-1/PDL1	PD-1 is expressed on activated T cells, B cells, and monocytes	[59]
	PD-L1 expression on proliferation and activation of T cells by interacting with PD-1.	[60]
	PD-1 negatively regulates the TCR signal by recruiting SHP-2 to the phosphorylated tyrosine residue in the cytoplasmic region	[61]
CTLA-4	Inhibition of T-cell proliferation, cell cycle progression, IL-2 production, and differentiation of T cells	[62]
CD40/CD40L	CD-40 is expressed mainly on B cells and regulation of B-cell proliferation	[63]
	CD40L expressed mainly on T cells	[64]
	T-cell-dependent B-cell differentiation and activation	[65]

Abbreviations: APCs, antigen-presenting cells; NK cells, natural killer cells; NKT cells, natural killer T cells; Tregs, T regulatory cells; DCs, dendritic cells; TCR, T-cell receptor; Th, T helper; MAP, Mitogen-activated protein kinase; STAT3, Signal transducer and activator of transcription 3; Tfh, T follicular helper cells; Tfr, T follicular regulatory cells; Tr1, type 1 regulatory T cells; SHP-2, src homology two-domain-containing tyrosine phosphatase 2.

## Abbreviations

APCs	Antigen-presenting cells
ARDs	Autoimmune rheumatic diseases
CIA	Collagen-induced arthritis
CRP	C-reactive protein
DCs	Dendritic cells
DM	Diabetes mellitus
FADD	Fas-associated death domain
ICPs	Immune checkpoints
Ig	Immunoglobulin
IL	Interleukin
MAP	Mitogen-activated protein kinase
MHC	Major histocompatibility complex
MMP	Metalloproteases
NFAT	Nuclear factor of activated T cells
NF-κB	Nuclear factor kappa B
NK cells	Natural killer cells
NKT cells	Natural killer T cells
RA	Rheumatoid arthritis
RF	Rheumatoid factor
SLE	Systemic lupus erythematosus
TCR	T-cell receptor
Tfh	T follicular helper cells
TGF-β	Transforming growth factor beta
Th	T helper
TLR	Toll-like receptor
TNF	Tumoral necrosis factor
Tregs	T regulatory cells
